# Supplementary material for: Change detection of auditory tonal patterns defined by absolute versus relative pitch information. A combined behavioural and EEG study
Source: PLoS One. 2021 Feb 25;16(2):e0247495. doi: 10.1371/journal.pone.0247495 (PMC7906474; doi:10.1371/journal.pone.0247495)
Supplement: Attachment — Submitted filename: SP2_responseReviewers.pdf [file pone.0247495.s004.pdf]

UNIVERSITÄT LEIPZIG

Faculty of Life Sciences
Institute of Psychology – Wilhelm Wundt

Leipzig University, Institute of Psychology – Wilhelm Wundt, 04109 Leipzig

Editor in Chief

PLOS ONE

Nina Coy
phone: +49 341 97-39575
nina.coy@uni-leipzig.de

Leipzig, 29 January 2021

Leipzig University

BioCog group

Neumarkt 9-19
04109 Leipzig
Germany

Web
https://home.uni-leipzig.de/biocog/

Manuscript revision PONE-D-20-25390

Dear Editor,

We are pleased to resubmit for publication the revised version of the manuscript entitled *“Change detection of auditory tonal patterns defined by absolute versus relative pitch information. A combined behavioural and EEG study.”* by Nina Coy, Maria Bader, Erich Schröger, and Sabine Grimm.

We appreciate the positive response to our first revision. We have amended the requested changes from Reviewer 1 as follows.

Reviewer #1: “The authors have thoroughly addressed all my comments. I believe the concern about separation of MMN and N2b is most adequately addressed in Fig 3 of the response to reviewers, which directly compares the ERPs in active and passive paradigms. This figure, and some of the preceding discussion, should be included in the paper itself as further evidence of the temporal overlap between MMN and N2b components in the absolute condition. I would also recommend making clearer that the ERPs shown in column 3 are difference waves, and which waveforms are from the active and which are from the passive condition, in both the legend and the caption. I eventually figured it out but it took me a while. Otherwise I think the paper is much better, much clearer, and is ready for publication.“

We have now included the, previously supplementary, figure comparing the ERP data between active and passive listening to the main manuscript body (now Fig 5) and extended its discussion. We changed the legend and added some more explicit information to improve its readability, especially concerning the differentiation between stimulus level and difference level ERPs.

Additionally, we ran all our figures through PACE and have uploaded the newly formatted figures to the editorial manager.

Yours sincerely,
Nina Coy (on behalf of all co-authors)
